# Using medication utilization information to develop an asthma severity classification model

**DOI:** 10.1186/s12911-017-0571-9

**Published:** 2017-12-20

**Authors:** Tsung-Hsien Yu, Pin-Kuei Fu, Yu-Chi Tung

**Affiliations:** 1 Department of Health Care Management, National Taipei University of Nursing and Science, No.89, Nei-Chiang St, Taipei, Taiwan; 2 Institute of Health Policy and Management, College of Public Health, National Taiwan University, No. 17, Xu-Zhou Rd, Taipei, Taiwan; 3 Department of Critical Care Medicine, Taichung Veterans General Hospital, No. 1650 Taiwan Boulevard Sect. 4, Taichung, Taiwan; 4 Department of Biotechnology, Hungkuang University, No. 1018, Taiwan Boulevard Sec. 6, Taichung, Taiwan

**Keywords:** Asthma, Claims data, Medication utilization, Severity classification model development

## Abstract

**Background:**

Claims data are currently widely used as source data in asthma studies. However, the insufficient information in claims data related to level of asthma severity may negatively impact study findings. The present study develops and validates an asthma severity classification model that uses medication utilization in Taiwan National Health Insurance claims data.

**Methods:**

The National Health Insurance Research Database was used for the years 2006–2012 and included a total of 7221 patients newly diagnosed with asthma in 2007 for model development and in 2008 for model validation. The medication utilization of patients during the first year after the index date was used to classify level of severity, and the acute exacerbation of asthma during the second through fourth years after the index date was used as the outcome variable. Three models were developed, with subjects classified into four, three, and two groups, respectively. The area under the receiver operating characteristic curve (AUC) and the Kaplan-Meier survival curve were used to compare the performances of the classification models.

**Results:**

In development data, the distribution of subjects and acute exacerbation rate among the stage 1 to stage 4 were: 62.71%, 5.54%, 22.79%, and 8.96%, and 8.17%, 9.55%, 11.97%, and 14.91%, respectively. The results also showed the higher severity groups to be more prone to being prescribed oral corticosteroids for asthma control, while lower severity groups were more likely to be prescribed short-acting medication and inhaled corticosteroid treatment. Furthermore, the results of survival analysis showed two-group classification was recommended and yield moderate performance (AUC = 0.671). In validation data, the distribution of subjects, acute exacerbation rates, and medication uses among stages were similar to those in development data, and the results of survival analysis were also the same.

**Conclusions:**

Understanding asthma severity is critical to conducting effective, scholarly research on asthma, which currently uses claims data as a primary data source. The model developed in the present study not only overcomes a gap in the current literature but also provides an opportunity to improve the validity and quality of claims-data-based asthma studies.

## Background

Asthma is a common, chronic disease involving inflammation of the small airway that affects more than 300 million people around the world [1]. An estimated 24.6 million Americans had asthma in 2015, including 8.4% of all children and 7.6% of all adults [2]. A 2017 estimate pegged the cost of asthma in the United States at $56 billion annually, with an additional $5.9 billion in productivity losses [3]. In Taiwan, asthma is also common, with an estimated prevalence rate of 12% of the total population [4]. The average hospitalization-related expenditures of asthmatic patients in Taiwan are 2.7 times that of other patient categories [5].

Using claims data to research asthma issues on a national or regional scale has become increasingly popular in recent years [6–8], with benefits frequently including lower costs, larger sample sizes, and easier longitudinal follow-up. However, this approach also presents several limitations and challenges in dealing with claims data such as insufficient code columns, validity issues (e.g. up-coding or down-coding to fit the payment scheme), and being unable to determine disease severity [9–11]. The latter is the most important factor that limits the use of claims data in related outcome research.

In order to resolve this limitation on determining disease severity, previous studies have used data related to medication utilization such as “controllers-to-total-asthma-drug” ratios and inhaled corticosteroids plus leukotriene antagonist receptors to estimate severity [12–15]. A recent systematic review study by Jacob et al. found 54 articles in the literature that had used claims data to assess asthma severity [16]. They further found that four types of algorithms were used to classify asthma severity, including Healthcare Effectiveness Data and Information Set (HEDIS) criteria, Leidy criteria, the Global Initiative for Asthma (GINA) criteria, Canadian Asthma Consensus Guidelines (CACQ). Of these, the HEDIS criteria were the most widely used.

All of the abovementioned algorithms use medical and medication utilizations to classify asthma severity in claims data. For example, the criteria of HEDIS include asthma-related patient data such as numbers of outpatients, admission, and ER visits and data on asthma medication dispensation [17, 18] but only classifies patients into persistent and intermittent parameters. The Leidy criteria rely on data on asthma medication dispensation only [19], with the frequency of oral corticosteroid prescriptions and short-acting β_2_-agonist used to classify level of severity into mildly persistent, moderately persistent, and severely persistent. However, information on model validation is insufficient for all of the models, with the exception of the CACQ [20].

The GINA guidelines are the most important reference for asthma treatment. These guidelines were published by an international organization that was launched in 1993 in collaboration with the National Heart, Lung, and Blood Institute, National Institutes of Health in the United States and the World Health Organization in order to develop a global strategy for managing and preventing asthma. GINA reports have provided the foundation for many national guidelines. They are prepared by international experts from primary, secondary, and tertiary care data and are updated annually following a review of the current evidence. Prior to 2014, GINA guidelines categorized asthma patients based on level of symptoms, of airflow limitation, and of lung function variability. The GINA guidelines were revised significantly in 2014 and now provide recommendations for categorizing levels of asthma control. In addition, most of the current severity classification models were developed before 2011. To the best of the knowledge of the present authors, no study in the literature has used current GINA severity classification criteria to develop an asthma severity classification model.

The Taiwan National Health Insurance (NHI) program, which currently covers roughly 23 million citizens and residents**,** was launched in 1995. Claims data, which are extracted from data collected regularly by the NHI**,** have been often used to explore the outcomes and quality of healthcare with regard to many diseases, including asthma [7, 8, 21–23]. However, as noted above, the classification of asthma severity in Taiwan NHI claims data remains an unresolved issue. Therefore, the purpose of the present study is to use the most updated GINA guidelines as a reference to develop an asthma severity classification model using Taiwan NHI claims data and to validate the feasibility of this model.

## Methods

### Data sources

The present study employed a retrospective, database cohort study and used data in the Taiwan National Health Insurance Research Database (NHIRD) from the time period 2006 to 2012. In Taiwan, the National Health Insurance Administration is the sole insurer and has implemented national health insurance (NHI) since March 1, 1995. The NHIRD covers the 23 million enrollees in the NHI program (approximately 99.9% of Taiwanese citizens), and almost all healthcare facilities are NHI-contracted providers. The NHI claims data includes inpatient medical benefit claims, ambulatory care medical benefit claims, pharmaceutical benefit claims, contracted medical care institutions, health professionals in contracted medical care institutions, and beneficiaries.

### Ethics statement

The study protocol was fully reviewed and approved by the Ethics Committee for Clinical Research, National Taiwan University Hospital (Taipei, Taiwan; protocol # 201601056RINB). The dataset was obtained from the NHIRD and all personal identification information has been encrypted. Therefore, written informed consent was not necessary.

### Patient population

**Fig. 1:**
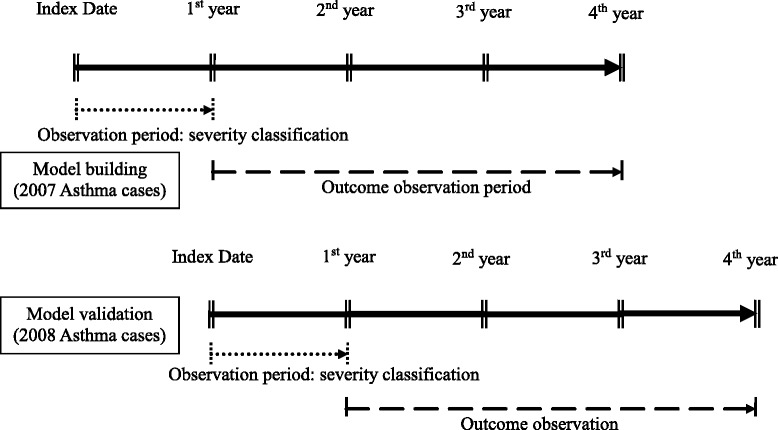
Data collection procedure

A retrospective study design was conducted. The medication utilization of patients during the first year after the index date was used to classify level of severity, and the acute exacerbation of asthma during the second through fourth years after the index date was used as the outcome variable (Fig. 1). The ambulatory, inpatient, and enrollment data of the asthma cohort were extracted from the NHIRD for the year 2007 in order to identify all patients with “newly diagnosed asthma in the year of 2007”. The date of the first diagnosis of asthma was used as the index date, and patients with two or more outpatient service claims or one or more inpatient care claim with a primary or secondary diagnosis of asthma (International Classification of Diseases, Ninth Revision, Clinical Modification [ICD-9-CM] codes including 493.0, 493.1, 493.2, 493.8 and 493.9) during 2007 were included as subjects [7, 8, 21]. Otherwise qualified patients who had been diagnosed with asthma prior to 2007 were excluded. Patients were also excluded if they: (1) were <18 years of age, (2) had withdrawn from coverage during the observation period, or (3) had been diagnosed with chronic obstructive pulmonary disease (COPD) prior to the index date. Additionally, for model validation, the population of asthmatics newly diagnosed in 2008 was recruited using the same inclusion and exclusion criteria.

### Model development and validation of the asthma severity classification model

**Fig. 2:**
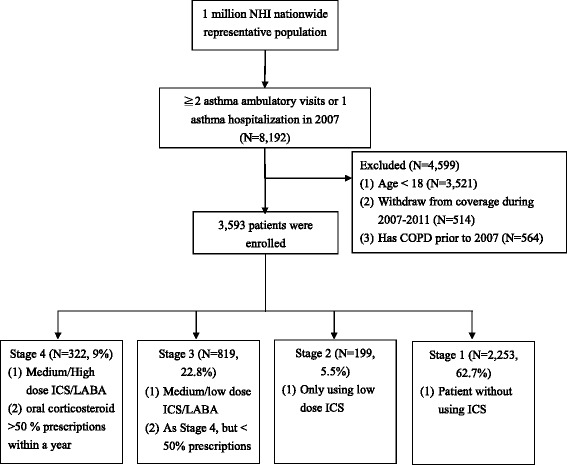
Flow diagram of patient selection and severity classification

The classification criteria that were used in the present study referred to Reliever/Oral Steroid Use (ROSU) [24] and GINA 2014 guidelines [25]. Although GINA classifies severity into five steps, data on Immunoglobulin E (IgE) utilization are not available in Taiwan NHI claims data. Thus, an expert meeting was held to discuss how to group patients and determine the criteria. Ultimately, the present study chose to classify asthma severity into four groups according to the following procedure and criteria: 1) A subject was classified as stage 4 (GINA step 4–5) if at least 50% of her/his prescriptions during the target year included medium/high dose-inhaled corticosteroid/long-acting inhaled β_2_-agonist (ICS/LABA) combinations or oral corticosteroids (OCSs); 2) A subject was classified as stage 3 (GINA step 3) if she/he had prescriptions of medium/low dose ICS/LABA or <50% of prescriptions included medium/high-dose ICS/LABA during the target year; 3) A subject was classified as stage 2 (GINA step 2) if she / he received only low-dose ICS prescriptions during the target year; and 4) All other patients were classified as stage 1 (GINA step 1) (Fig. 2).

Moreover, the event of acute exacerbation was used as the gold standard, including: (1) had an asthma-related hospitalization and at least one ER visit and (2) received two or more than two consecutive short-acting medication prescriptions within a 10-day period. We also collected information on subject age, gender, score on the Charlson Comorbidity Index, obesity status, sinusitis status, and Gastroesophageal Reflux Disease (GERD) status and used these data as covariates.

Three models were developed in the present study. In model 1, asthma severity was classified into four groups (stages 1 to 4); in model 2, asthma severity was classified into three groups (stages 1–2, 3, and 4); and in model 3, asthma severity was classified into two groups (stages 1–2 and 3–4). All of the covariates were included in all of the models. As mentioned above, newly diagnosed asthmatic patients in 2007 were selected for model development, while newly diagnosed asthmatic patients in 2008 were selected for model validation.

### Statistical analysis

All of the analyses were conducted using SAS statistical software, version 9.4 (SAS Institute Inc., Cary, NC) and the statistical level of significance was set at 0.05. Descriptive statistics were presented as number, mean, and standard deviation (SD) for continuous variables and as frequency, and percentage for categorical variables. Differences were examined using the Chi-square test for categorical variables and the analysis of variance (ANOVA) test for continuous variables. Kaplan-Meier survival analysis was performed, with the differences in survival functions between severity groups assessed using the log rank test. The area under the receiver operating characteristic (ROC) curve (AUC) was used to compare the performances between various classification models. Additionally, the multivariable Cox proportion hazard regression was used to examine the effect of asthma severity on the acute exacerbation of asthma after adjusting for the covariates.

## Results

Table 1 illustrates the characteristics, medication, and medical utilization of the development group. A total of 3593 patients were included as subjects. Three-fifths (58.47%; 2101) were female and around 60% were above 45 years of age, with the largest numbers in the >65 (22.96%) and 18–34 (24.49%) years-of-age groups. In terms of comorbidity, the mean score for the Charlson Comorbidity Index (CCI) was 0.39, which included 10, 40, and 11 subjects who had been diagnosed with obesity, sinusitis, and gastroesophageal reflux disease, respectively. In terms of medication utilization, 866 (24.10%) received at least one ICS/LABA combination prescription, 822 (22.88%) received at least one short-acting beta agonist (SABA) prescription, 366 (10.19%) received at least one oral corticosteroid prescription, 309 (8.60%) received at least one ICS prescription, 111 (3.09%) received at least one short-acting muscarinic receptor antagonist (SAMA) prescription, and 155 (4.31%) received at least one SABA and SAMA combination prescription within one year of the index date. In terms of acute exacerbation, 349 (9.71%) had experienced at least one acute exacerbation event, 52 (1.45%) had been admitted to hospital, 160 (4.45%) made an ER visit, and 218 (6.07%) had received ≥2 consecutive short-acting medication prescriptions within a 10-day period.

**Table 1:** Subject demographic characteristics and severity classifications: development data

Variables	All asthma, (*n* = 3593)	Classified by Asthma severity, n (%)				*p*-value
		Stage 1 (*n* = 2253, 62.71%)	Stage 2 (*n* = 199, 5.54%)	Stage 3 (*n* = 819, 22.79%)	Stage 4 (*n* = 322, 8.96%)	
Gender, n(%)						0.1548
Female	2101 (58.47)	1285 (57.04)	123 (61.81)	498 (60.81)	195 (60.56)	
Male	1492 (41.53)	968 (42.96)	76 (38.19)	321 (39.19)	127 (39.44)	
Age, n(%)						<.0001
18–34	880 (24.49)	511 (22.68)	55 (27.64)	231 (28.21)	83 (25.78)	
35–44	609 (16.95)	355 (15.76)	46 (23.12)	143 (17.46)	65 (20.19)	
45–54	660 (18.37)	406 (18.02)	39 (19.60)	148 (18.07)	67 (20.81)	
55–64	619 (17.23)	405 (17.98)	31 (15.58)	144 (17.58)	39 (12.11)	
≧65	825 (22.96)	576 (25.57)	28 (14.07)	153 (18.68)	68 (21.12)	
CCI, Mean(SD)	0.39 (0.85)	0.41 (0.85)	0.28 (0.84)	0.34 (0.73)	0.38 (1.05)	0.0435
Obesity, n(%)	10 (0.28)	9 (0.40)	0 (0.00)	1 (0.12)	0 (0.00)	0.6141
Sinusitis, n(%)	40 (1.11)	21 (0.93)	2 (1.01)	15 (1.83)	2 (0.62)	0.1808
GERD, n(%)	11 (0.31)	10 (0.44)	0 (0.00)	0 (0.00)	1 (0.31)	0.2207
Medication utilization, n(%)						
ICS/LABA	866 (24.10)	0 (0.00)	0 (0.00)	660 (80.59)	206 (63.98)	<.0001
ICS	309 (8.60)	0 (0.00)	199 (100.0)	89 (10.87)	21 (6.52)	<.0001
OCS	366 (10.19)	0 (0.00)	0 (0.00)	203 (24.79)	163 (50.62)	<.0001
SABA	822 (22.88)	423 (18.77)	54 (27.14)	267 (32.60)	78 (24.22)	<.0001
SAMA	111 (3.09)	49 (2.17)	5 (2.51)	43 (5.25)	14 (4.35)	0.0001
SABA/SAMA	155 (4.31)	71 (3.15)	9 (4.52)	56 (6.84)	19 (5.90)	<.0001
# of patient having acute exacerbation, n(%)	349(9.71)	184(8.17)	19(9.55)	98(11.97)	48 (14.91)	0.0001
Hospitalization	52 (1.45)	28 (1.24)	4 (2.01)	9 (1.10)	11 (3.42)	0.0144
Emergency department visit	160(4.45)	79(3.51)	8(4.02)	54(6.59)	19(5.90)	0.0016
Short-acting drug	218(6.07)	115(5.10)	9(4.52)	63(7.69)	31(9.63)	0.0015

*CCI* Charlson Comorbidity Index, *GERD* Gastroesophageal reflux disease, *ICS* inhaled corticosteroid, *OCS* oral corticosteroid, *SABA* short-acting inhaled β_2_-agonist, *LABA* long-acting inhaled β_2_-agonist, *SAMA* short-acting muscarinic receptor antagonists

According to the algorithm, the distribution of subjects among the four stages were: stage 1–2253 (62.71%), stage 2–199 (5.54%), stage 3–819 (22.79%), and stage 4–322 (8.96%), respectively. Further, Table 1 shows comparisons of patient characteristics, medication utilization, and acute exacerbation events among subjects in the four groups. The results revealed that patient age, medication, and acute exacerbation events showed significant differences among these groups. The higher severity groups had a slightly higher proportion of older individuals. In terms of medication utilization, the data also showed that higher severity groups (stages 3–4) were more likely to receive prescriptions of OCS for asthma control. In contrast, the lower severity groups were primarily prescribed SABAs, SAMAs, and ICSs. Further, the percentage of acute exacerbation events increased with level of severity.

Table 2 shows the results of the descriptive analysis of the validation group, which included a total of 3628 subjects. In terms of patient characteristics, the results are similar to the development group. Regarding medication utilization, in general, the patterns of medication utilization are almost the same between the two groups (development and validation), with the exception of the slightly higher utilization of SABA in the stage-3 validation group. In terms of acute exacerbation, overall acute exacerbation was around 10%, which is similar to the development group, although the percentage of acute exacerbation events increased with level of severity, with stage 3 rather than stage 4 showing the highest rate of acute exacerbation.

**Table 2:** Subject demographic characteristics and severity classifications: validation data

Variables	All asthma, (*n* = 3628)	Classified by Asthma severity, *n* (%)				*p*-value
		Stage 1 (*n* = 2276, 62.73%)	Stage 2 (*n* = 230, 6.34%)	Stage 3 (*n* = 807, 22.24%)	Stage 4 (*n* = 315, 8.68%)	
Gender, n (%)						0.7073
Female	2107 (58.08)	1309 (57.51)	141 (61.30)	472 (58.49)	185 (58.73)	
Male	1521 (41.92)	967 (42.49)	89 (38.70)	335 (41.51)	130 (41.27)	
Age, n (%)						<.0001
18–34	874 (24.09)	503 (22.10)	72 (31.30)	221 (27.39)	78 (24.76)	
35–44	586 (16.15)	346 (15.20)	40 (17.39)	145 (17.97)	55 (17.46)	
45–54	686 (18.91)	430 (18.89)	48 (20.87)	150 (18.59)	58 (18.41)	
55–64	588 (16.21)	371 (16.30)	41 (17.83)	123 (15.24)	53 (16.83)	
≧65	894 (24.64)	626 (27.50)	29 (12.61)	168 (20.82)	71 (22.54)	
CCI, Mean (SD)	0.41 (0.86)	0.44 (0.88)	0.30 (0.69)	0.38 (0.86)	0.35 (0.76)	0.0252
Obesity, n (%)	18 (0.50)	12 (0.53)	0 (0.00)	4 (0.50)	2 (0.63)	0.8377
Sinusitis, n (%)	35 (0.96)	21 (0.92)	4(1.74)	7(0.87)	3(0.95)	0.5970
GERD, n (%)	10 (0.28)	6 (0.26)	0 (0.00)	4 (0.50)	0 (0.00)	0.5579
Medication utilization, n (%)						
ICS/LABA	853 (23.51)	0 (0.00)	0 (0.00)	658 (81.54)	195 (61.90)	<.0001
ICS	331 (9.12)	0 (0.00)	227 (98.70)	90 (11.15)	14 (4.44)	<.0001
OCS	358 (9.87)	0 (0.00)	0 (0.00)	188 (23.30)	170 (53.97)	<.0001
SABA	870 (23.98)	428 (18.80)	82 (35.65)	299 (37.05)	61 (19.37)	<.0001
SAMA	133 (3.67)	66 (2.90)	9 (3.91)	46 (5.70)	12 (3.81)	0.0040
SABA/SAMA	119 (3.28)	51 (2.24)	9 (3.91)	46 (5.70)	13 (4.13)	<.0001
# of patient having acute exacerbation, n (%)	361 (9.95)	186 (8.17)	21 (9.13)	118 (14.62)	36 (11.43)	<.0001
Hospitalization	59 (1.63)	26 (1.14)	1 (0.43)	21 (2.60)	11 (3.49)	0.0007
Emergency department visit	174 (4.80)	83 (3.65)	11 (4.78)	66 (8.18)	14 (4.44)	<.0001
Short-acting drug	230 (6.34)	121 (5.32)	11 (4.78)	74 (9.17)	24 (7.62)	0.0008

*CCI* Charlson Comorbidity Index, *GERD* Gastroesophageal reflux disease, *ICS* inhaled corticosteroid, *OCS* oral corticosteroid, *SABA* short-acting inhaled β_2_-agonist, *LABA* long-acting inhaled β_2_-agonist, *SAMA* short-acting muscarinic receptor antagonists

**Fig. 3:**
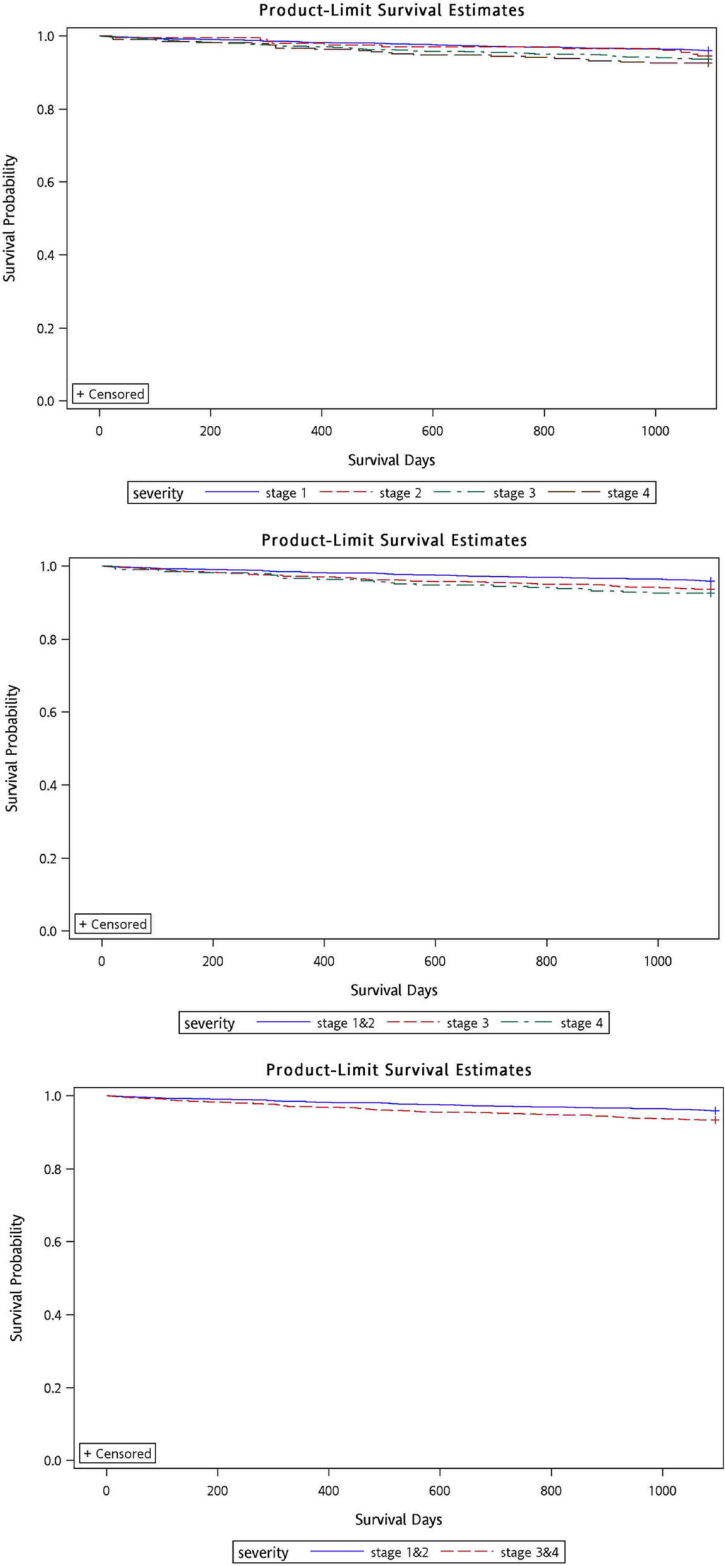
Results of Kaplan-Meier survival analysis

Figure 3 presents the results of the Kaplan–Meier survival analysis. All of the results achieved statistical significance (*p* < 0.001) regardless of which severity classification model was applied. Nevertheless, the survival curves of stage 4 and stage 3 were crossed, when both Model 1 (four severity groups) and Model 2 (three groups) were applied. Therefore, Models 1 and 2 were excluded. In terms of classification performance, the area under the curve of Model 3 was 0.671. For model validation, the same inclusion and exclusion criteria were used to select newly diagnosed asthma in 2008 and the same classification algorithms were used to classify asthma severity. The classification performance also showed that the area under the curve of Model 3 was 0.702. The results are thus similar between the development and validation groups, suggesting that the developed model is stable. Finally, the numbers of censored subjects among models are presented in Table 3, and Table 4 shows the results of the Cox proportional hazard model and revealed that the more severe groups faced a higher risk of acute exacerbation.

**Table 3:** The numbers of censored subjects, by model

	Severity	Initial numbers	Number of censored subjects						
			<200 days	201–400 days	401–600 days	601–800 days	801–1000 days	>1000 days	Total
M1	1	2253	50	38	24	34	24	14	184
	2	199	1	5	4	0	4	5	19
	3	819	28	16	20	16	9	9	98
	4	322	13	12	9	5	8	1	48
M2	1	2452	51	43	28	34	28	19	203
	2	819	28	16	20	16	9	9	98
	3&4	322	13	12	9	5	8	1	48
M3	1&2	2452	51	43	28	34	28	19	203
	3&4	1141	41	28	29	21	17	10	146

**Table 4:** Results of Cox proportional hazard models

	Model 1 (4 groups)		Model 2 (3 groups)		Model 3 (2 groups)	
	HR(95%CI)	*p*-value	HR(95%CI)	*p*-value	HR(95%CI)	*p*-value
Asthma severity						
Stage 1	Reference		Reference		Reference	
Stage 2	1.389(0.741–2.603)	0.3053				
Stage 3	1.597(1.133–2.252)	0.0075	1.547(1.105–2.165)	0.0109	1.634(1.211–2.203)	0.0013
Stage 4	1.916(1.220–3.010)	0.0047	1.857(1.189–2.900)	0.0065		

*HR* hazard ratio, *CI* confidence interval

After adjusted for age, gender, Charlson Comorbidity Index, obesity, Sinusitis, and Gastroesophageal reflux disease

## Discussions

Asthma is a chronic airway inflammatory disorder that affects more than 300 million people worldwide and causes substantial morbidity for patients as well as economic loss for society [26]. Many of these studies use claims data for research and analysis. However, the lack of information on asthma severity in claims data is a major limitation that may diminish the value of research findings. In this study, we referred to ROSU and 2014 GINA guidelines to develop asthma severity classification models in claims data. After model development and validation, the results of the present study support the validity of using the medication utilization information in claims databases to classify asthma patients into two groups. Thus, the achievements of the present study may help fill this gap and lead to academic advances.

The major purpose of claims data is for reimbursement, therefore, when it is being applied to other purposes, using existing information to find out proxy indicators is necessary and important. For example, risk adjustment is an important procedure for healthcare organization comparison, and the information of disease severity could be the most essential component included. Recently, a lot of studies tried to use the information of medication and healthcare utilization to classify disease severity (e.g. stroke [27], COPD [9]), and outcomes of care (e.g. surgical site infection [28]). Therefore, the medication and healthcare utilization could be a good source of information, and might be also more accurate than ICD codes.

In addition, this study highlighted several issues that are worth further discussion. First, regarding necessity for developing a new model to classify asthma severity in claims data instead of adopting an existing model, several other models have been developed, and prior research also used the information of medication and healthcare utilization as criteria. However, the model that was developed in the present study presents several unique advantages, including: (1) the present sample was abstracted from a nationwide database. Thus, the representativeness of this sample should be superior to existing models and (2) the procedure used to develop the model in the present study is more comprehensive than those used in previous studies. In addition to referencing existing experience, the authors convened an expert meeting to confirm the feasibility and practicality of the selected classification criteria and also used data from another year for model validation.

Second, regarding the explicitness of severity classification criteria, although the existing models have provided many criteria to implement, they were developed many years ago and the appropriateness of these criteria should be reviewed carefully. Besides, existing models and criteria faced certain limitations in implementation. One example is whether it is appropriate to use an absolute number as a cutoff point. A physician-ordered modification to a prescription may not relate to a change in patient severity level. Therefore, the present study used the proportion of medium/high-dose ICS or ICS/LABA in order to avoid this limitation, while the expert meeting helped make the developed model more applicable in daily practice.

Third, concerning the time lag between the years of data and the severity classification standards used to test the developed model, although the year of data we used and the year of severity classification standard we adopted were not consistent, but the medications and treatment medications were similar with data collected over the past decade, with the exception of IgE. Only the severity classification standard had been revised over time [25, 29]. Therefore, the inconsistency between the year of data used and the year of severity classification standard may not pose a significant limitation or problem for the present study.

Claims data is widely used for various purposes, but lacking of the information of disease severity is the major defeat. Based on the experience of this study, researchers can follow the model development procedure to develop and validate disease severity classification models for other diseases, especially in chronic disease. Researchers can pay more attention to disease severity classification model development, and policy makers also can apply them to optimize local healthcare delivery.

### Limitations

Although we followed rigorous procedures in developing and validating the asthma severity classification models in the claims data, the present study is affected by several limitations. Most importantly, information on actual asthma severity level and the distribution of severity among asthma patients was not directly obtainable from claims data. In order to minimize the impact of this limitation, we selected acute exacerbation of asthma to validate our model. The acute exacerbation of asthma is highly correlated with asthma severity level [26]. Thus, this limitation could be alleviated. Next, with regard to the rate of guideline adherence, several studies have indicated that poor adherence to guidelines is an issue in asthma care [30, 31]. Moreover, our data were extracted from a period prior to the GINA guidelines major revision in 2014. Therefore, physician prescription patterns should have changed after the 2014 GINA guidelines were published. However, medication utilization may be the only information available to classify asthma patient severity in claims data. Therefore, an expert meeting was convened in order to reduce the effect of low guideline adherence. Nevertheless, this limitation remains difficult to avoid. Finally, unmeasurable factors such as air pollution, allergens, and other environmental factors were not available in the present study, which may cause the value of the area under the curve was lower than 0.7.

## Conclusion

Accurately understanding level of asthma severity is necessary and critical to asthma research. Current studies widely use claims data to collect the data necessary to assess asthma severity. The results of this study suggest that it is possible to use the medical utilization of patients to classify asthma severity in claims data. The model developed in the present study has the potential not only to improve the validity and quality of asthma research but also to analyze the data and explain the results in advance.

## Abbreviations

CACQ: Canadian Asthma Consensus Guideline
GINA: Global Initiative for Asthma
HEDIS: Healthcare Effectiveness Data and Information Set
ICS: Inhaled corticosteroids
LABA: Long-acting inhaled β2-agonist
NHI: National Health Insurance
OCS: Oral corticosteroid
SABA: Short-acting inhaled β2-agonist
SAMA: Short-acting muscarinic receptor antagonists
